# Downregulation of N^6^-methyladenosine binding YTHDF2 protein mediated by miR-493-3p suppresses prostate cancer by elevating N^6^-methyladenosine levels

**DOI:** 10.18632/oncotarget.23365

**Published:** 2017-12-18

**Authors:** Jiangfeng Li, Shuai Meng, Mingjie Xu, Song Wang, Liujia He, Xin Xu, Xiao Wang, Liping Xie

**Affiliations:** ^1^ Department of Urology, First Affiliated Hospital, School of Medicine, Zhejiang University, Hangzhou, 310000, Zhejiang Province, China; ^2^ Department of Urology, Zhejiang Provincial People’s Hospital, Hangzhou, 310000, Zhejiang Province, China

**Keywords:** m6A, YTHDF2, miR-493-3p, epigenetics, PCa

## Abstract

Recent evidence suggests that m6A modifications regulate the progressions of several types of tumors. YTHDF2, an m6A reader, has been implicated in the regulation of hepatocellular carcinoma (HCC). miR-493-3p has been defined as tumor suppressor that inhibits the progressions of several types of cancers. However, the functions and mechanisms of YTHDF2 and the indirect m6A regulated role of miR-493-3p in prostate cancer (PCa) remains to be elusive. In this study, immuno-histochemical (IHC) staining and chromogenic in situ hybridization (CISH) were performed to find YTHDF2 was frequently upregulated but miR-493-3p was downregulated in both PCa tissues and cell lines (DU-145 and PC3) which was negatively correlated with each other. Knock down of YTHDF2 significantly elevated m6A levels, and inhibited the cell proliferation and migration of DU-145 and PC3 cell lines. The dual-luciferase reporter assay confirmed YTHDF2 as the direct target of miR-493-3p. In addition, forced expression of miR-493-3p consistently elevated the m6A levels and inhibited proliferation and migration with the knock down of YTHDF2. In contrast, overexpression of YTHDF2 and inhibition of miR-493-3p conversely reduced m6A levels. Additionally, the rescue experiments revealed that inhibition of miR-493-3p abrogated the suppression of proliferation and migration induced by si-YTHDF2. To conclude, YTHDF2 and miR-493-3p, as two crucial m6A regulators, are involved in the progression of PCa by indirectly modulating m6A levels. In view of these promising results, YTHDF2 and miR-493-3p may provide new insights into the carcinogenesis and new potential therapeutic targets for PCa.

## INTRODUCTION

Prostate cancer (PCa) is the most common cancer among men in developed countries, and holds the second highest mortality rate induced by cancer. More than one million cases are diagnosed annually, and the mortality burden has risen to over 300,000 deaths per year [1]. However, determining treatments for PCa is still challenging, especially for high-risk PCa and castration-resistant PCa (CRPC). Thus, further elucidation of the profound mechanisms involved in PCa progression is imperative.

Methylation of the N6 position of adenosine (m6A) is a post-transcriptional modification of RNA. Although m6A was initially discovered in 1974, its specific function and mechanisms are poorly understood [2]. Since the identification of first demethylase, FTO, confirmed that the m6A modification is reversible, studies about m6A have been surging [3]. With the discovery of the methyltransferase complex and several m6A-binding proteins, a basic understanding of the m6A modification has been established. In the reversible process of m6A modification, the methyltransferase ‘writer’ complex (METTL3, METTL14, WTAP, RBM15) catalyzes RNAs to promote and produce methylation at the N6 position of adenosine, then the methylated mRNAs are recognized and bound by different ‘reader’ proteins (YTHDF1/2/3, YTHDC1/2 and HNRNPA2B1) to initiate variable functions. Conversely, the ‘eraser’ protein FTO removes the written methylation to reverse the m6A modification. [4-8]. Previous studies have found that m6A is the most prevalent RNA modification and distributed in various RNA oligonucleotides. So far, m6A sites have been detected in approximately 7600 mRNA transcripts, and more than 300 non-coding RNAs in humans [9, 10]. In addition, m6A sites were found to be highly abundant near stop codons in 3’-UTR and within internal long exons [11].

Although, the components of m6A modification machinery and the distributions of m6A are known, the specific functions and mechanisms involved are uncharacterized and remain elusive. With the continuous investigations of m6A functions and mechanisms, emerging evidence has indicated that the m6A modification, as a novel epigenetic regulator, may be involved in various physiological processes and diseases, including circadian rhythms, yeast meiosis, embryonic stem cell self-renewal and maternal-to-zygotic transition (MZT), and especially the carcinogenesis of several tumors [12-15]. In addition, increasing studies have confirmed that m6A may possibly regulate cancers, however, m6A levels varied depending on cancer types. ALKBH5 and m6A depletion was reported to induce breast cancer via ALKBH5-mediated NANOG mRNA demethylation [16]. Similarly, METTL14 suppressed the metastatic potential of HCC by modulating m6A-dependent tumor suppressor primary miRNA processing [17]. However, in acute myeloid leukemia (AML), different m6A-associated proteins exhibited the various effects. FTO plays an oncogenic role by reducing the m6A levels of two tumor suppressors (ASB2 and RARA), thus, leading to the downregulation of these two genes [18]. But analysis of The Cancer Genome Atlas reveals METTL3, METTL14, and RBM15 are all upregulated in AML. In addition, RBM15 plays a vital role in maintaining quiescence in hematopoietic stem cells and in megakaryocyte leukemia cell line differentiation by controlling the splicing of key differentiation genes (GATA1, RUNX1, TAL1, and c-MPL) [19, 20]. To conclude, the m6A modification is indeed involved in the progression of several tumors, however, the differing m6A levels and various m6A-associated proteins may resulted in diverse even opposite outcomes in variable cancers. YTHDF2, a member of the YTH domain family was the first m6A reader protein to be discovered. After the binding of YTHDF2 to m6A sites, the recognized mRNAs were degraded and m6A levels were subsequently reduced [21, 22]. YTHDF2 was found to regulate m6A levels in HCC [23], however, its expression pattern, functions and mechanisms in major tumors especially in PCa remain elusive.

As one of the noncoding RNAs, microRNAs (miRNAs) are novel gene regulators that target the 3’-UTR of downstream mRNAs to accelerate their degradation and/or block their translations via seed region matching. miR-493-3p was transcribed from the DLK1-DIO3 genomic region, which has been reported to act as a tumor suppressor in several cancers (lung cancer, lung cancer, bladder cancer and head and neck cancer) by targeting different oncogenes [24-27]. However, the expression pattern and specific functions of miR-493-3p, especially its m6A-regulated role in PCa was still unclear.

In this study, we are first to investigate the expression pattern of YTHDF2, and how it functions, in combination with the upstream factor miR-493-3p, to modulate PCa progression in a m6A modification way. Interestingly, we found that YTHDF2 was upregulated in both PCa cell lines and tissues. Downregulation of YTHDF2 significantly inhibited the proliferation and migration of PCa by elevating m6A levels. Moreover, miR-493-3p was identified as the direct upstream factor of YTHDF2 in suppressing the proliferation and migration of PCa cell lines. To conclude, the downregulation of YTHDF2 mediated by miR-493-3p significantly inhibited PCa progression by elevating m6A levels.

## RESULTS

### YTHDF2 is upregulated in PCa and negatively correlated with miR-493-3p

**Figure 1:**
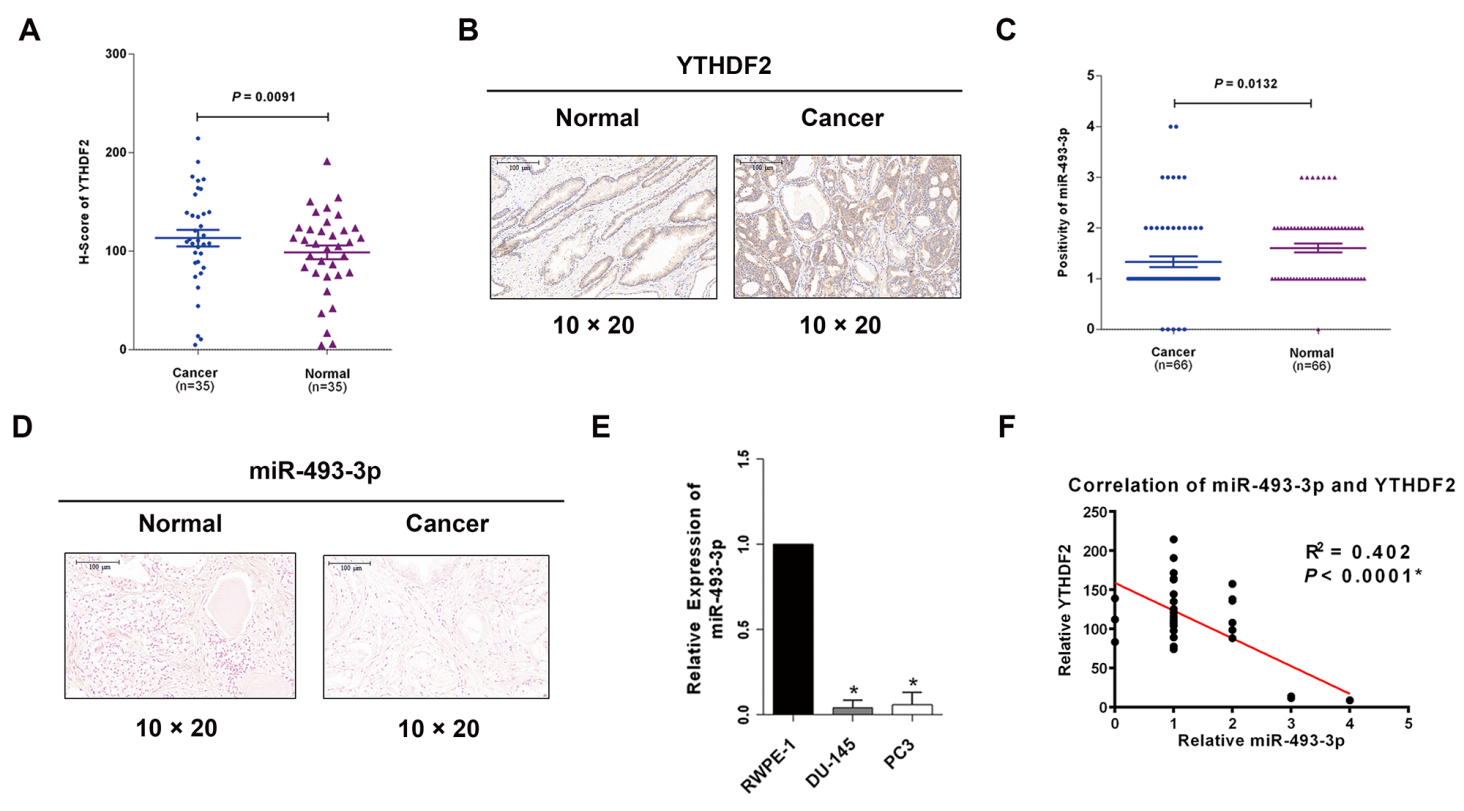
YTHDF2 is upregulated in PCa tissues and cell lines and negatively correlated with miR-493-3p **(A)** IHC of YTHDF2. The results of IHC demonstrated that YTHDF2 was upregulated in PCa tissues than adjacent normal tissues. **(B)** The representative images of IHC. YTHDF2 protein was located in the cytoplasm and nucleus in PCa. **(C)** CISH of miR-493-3p. The results of CISH revealed miR-493-3p was downregulated in PCa. **(D)** The representative images of CISH of miR-493-3p. **(E)** q-RT-PCR. miR-493-3p was downregulated in PCa cell lines (DU-145, PC3) compared with normal prostate cell line (RWPE-1). **(F)** Statistical analysis indicated a significant negative correlation between YTHDF2 and miR-493-3p. Error bars represent the S.E. obtained from three independent experiments; ^*^; *P* < 0.05. Scale bar = 100 μm.

Emerging evidence has indicated that global mRNA m6A level is associated with cancer. As an m6A reader, YTHDF2 triggered mRNA degradation by recognizing and binding to the written m6A sites of targeted mRNAs, which was also found to be involved in HCC [21-23]. However, its specific expression pattern and functions in PCa are still unclear. To primarily investigate the level of YTHDF2 in PCa, database (Oncomine, https://www.oncomine.org/resource/login.html) was explored to find YTHDF2 was always upregulated in three studies (Supplementary Figure 1). Surprisingly, in this study we found YTHDF2 was also significantly upregulated in PCa tissues with IHC staining in TMAs (*P* = 0.0091, Figure 1A) and was located in the cytoplasm and nucleus in PCa cells (Figure 1B). Besides, the clinical characteristics of the PCa patients are all listed in Table 1 which showed that high expression of YTHDF2 indicated a high tumor grade (Gleason score ≥ 7) (*P* = 0.034), while miR-493-3p revealed an opposite outcome (*P* = 0.035). miR-493-3p was previously reported to regulate the progression of various tumors. However, its role in PCa has not been studied. CISH was performed, and miR-493-3p was found to be downregulated in PCa (Figure 1C and 1D, *P* =0.0132). To Further detect the expression of miR-493-3p expression in two PCa cell lines (DU-145, PC3), q-RT-PCR was conducted and the results revealed that miR-493-3p markedly downregulated in both PCa cell lines compared to the normal prostate cell line (RWPE-1) (Figure 1E). Moreover, statistical analysis showed the negative correlation between miR-493-3p and YTHDF2 (Figure 1F, R2 = 0.402, *P* < 0.0001). The above results indicated the potential effects of YTHDF2 and miR-493-3p in regulation of PCa carcinogenesis.

**Table 1:** Clinical characteristics of the prostate cancer patients

	Expression of YTHDF2				
	Low		High		
	No. of patients	%	No. of patients	%	*P*
Total	16		19		
Age					
< 68	8	50.0	6	31.6	0.223
≥ 68	8	50.0	13	68.4	
**Tumor grade**^a^					
Low	10	62.5	5	26.3	0.034
High	6	37.5	14	73.7	
miR-493-3p					
High	8	50.0	3	15.8	0.035
Low	8	50.0	16	84.2	

^**a**^ Tumor grade: low: Gleason score < 7, high: Gleason score ≥ 7.

### Knock-down of YTHDF2 significantly upregulates global mRNA m6A levels to suppress PCa cell proliferation and migration

**Figure 2:**
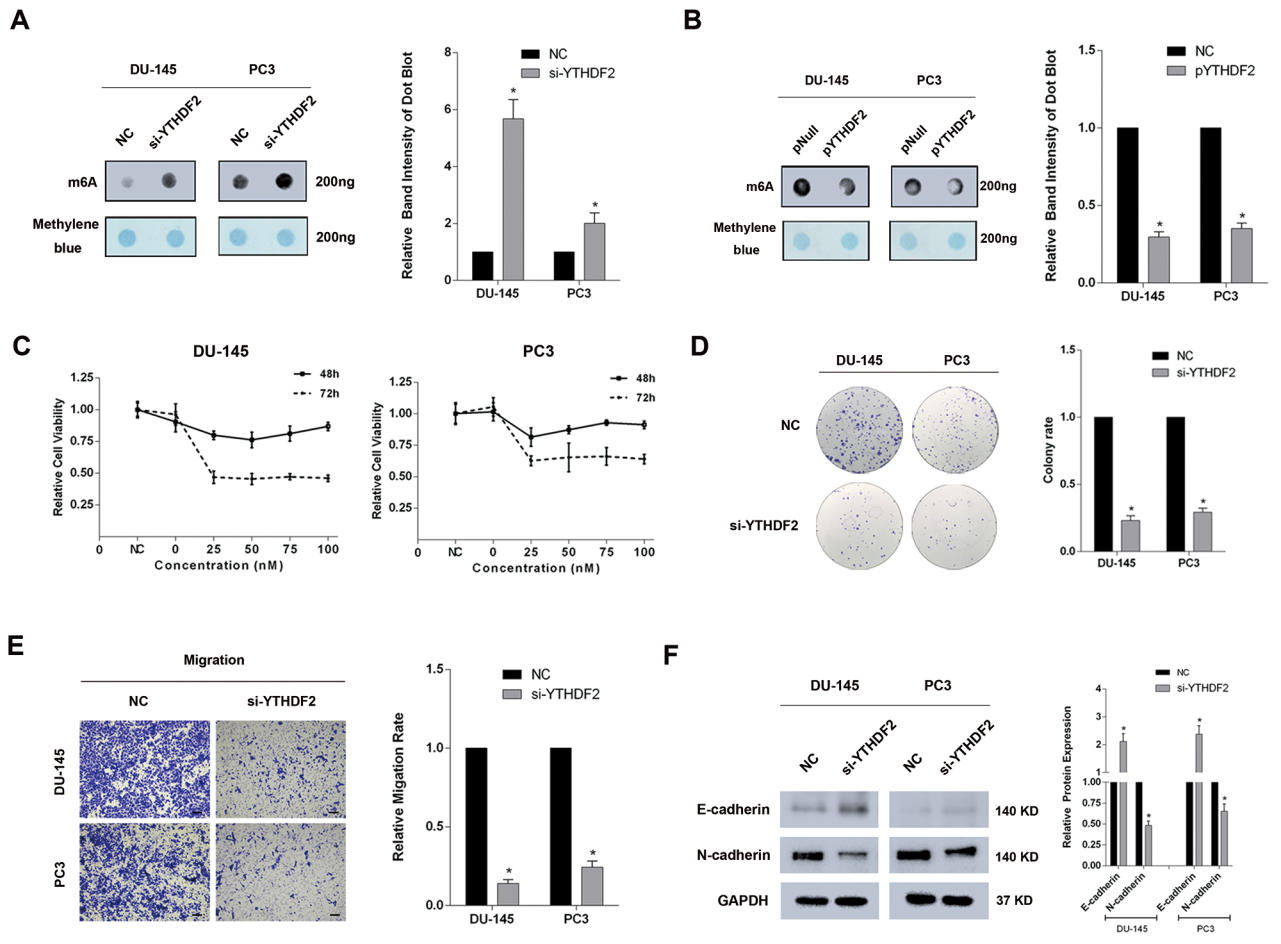
Knock-down of YTHDF2 significantly elevates the global mRNA m6A levels and inhibits proliferation and migration of PCa cell lines **(A)** m6A dot-blot assay. A significant elevation of m6A levels in DU-145 and PC3 cell lines transfected with si-YTHDF2 was detected. And the band intensity was measured and the result was shown behind. **(B)** m6A dot-blot assay. A significant reduction of m6A levels in DU-145 and PC3 cell lines transfected with pYTHDF2. And the band intensity was measured and the result was shown behind. **(C)** CCK-8 test. A significant suppression of proliferation at 48h and 72h was observed in DU-145 and PC3 cell lines transfected with si-YTHDF2. **(D)** Colony formation assay. Knock-down of YTHDF2 inhibited colony formation ability in DU-145 and PC3 cell lines. And the migration rate was calculated and shown behind. **(E)** Trans-well assay. Knock -down of YTHDF2 inhibited the migration in DU-145 and PC3 cell lines. And the migration rate was calculated and shown behind. **(F)** Western blot assay. A significant upregulation of E-cadherin and downregulation of N-cadherin was observed at protein level. And the band intensity of proteins was measured and the result was shown behind. Error bars represent the S.E. obtained from three independent experiments; ^*^; *P* < 0.05. Photographs of trans-well assay were obtained under 10x objective, and the scale bar = 100 μm.

To avoid off-targets effects, three siRNAs were merged together as si-YTHDF2-pool to transfect DU-145 and PC3 cell lines, and the high knock-down efficiency was confirmed at both mRNA and protein levels (Supplementary Figure 2). An m6A dot-blot assay was performed to investigate the global mRNA m6A levels. In addition, a significantly elevated global mRNA m6A level was observed in the YTHDF2 knock-down experiment (Figure 2A). In contrast, forced expression of YTHDF2 (pYTHDF2) reduced the global mRNA m6A levels in DU-145 and PC3 cell lines (Figure 2B). And a good overexpression efficiency was detected at protein level (Supplementary Figure 3). To determine the specific functions, a CCK-8 test was conducted and the data indicated the obvious inhibition of cell proliferation after knock-down of YTHDF2, likewise, the colony formation ability was also suppressed in DU-145 and PC3 cell lines treated with si-YTHDF2 (Figure 2C and 2D). To evaluate the migration rate, trans-well assays were performed, and an impairment of migration rate was observed in si-YTHDF2 transfected PCa cell lines (Figure 2E). Western blot analysis showed significant variations in the levels of EMT-associated proteins. (upregulation: E-cadherin, downregulation: N-cadherin) after si-YTHDF2 transfection in DU-145 and PC3 cell lines (Figure 2F). To conclude, as an important m6A reader, knock-down of YTHDF2 significantly suppressed the cell proliferation and migration by elevating the global mRNA m6A levels, which suggested the m6A modification and the reader protein YTHDF2 were involved in carcinogenesis of PCa.

### YTHDF2 is the direct target gene of miR-493-3p

**Figure 3:**
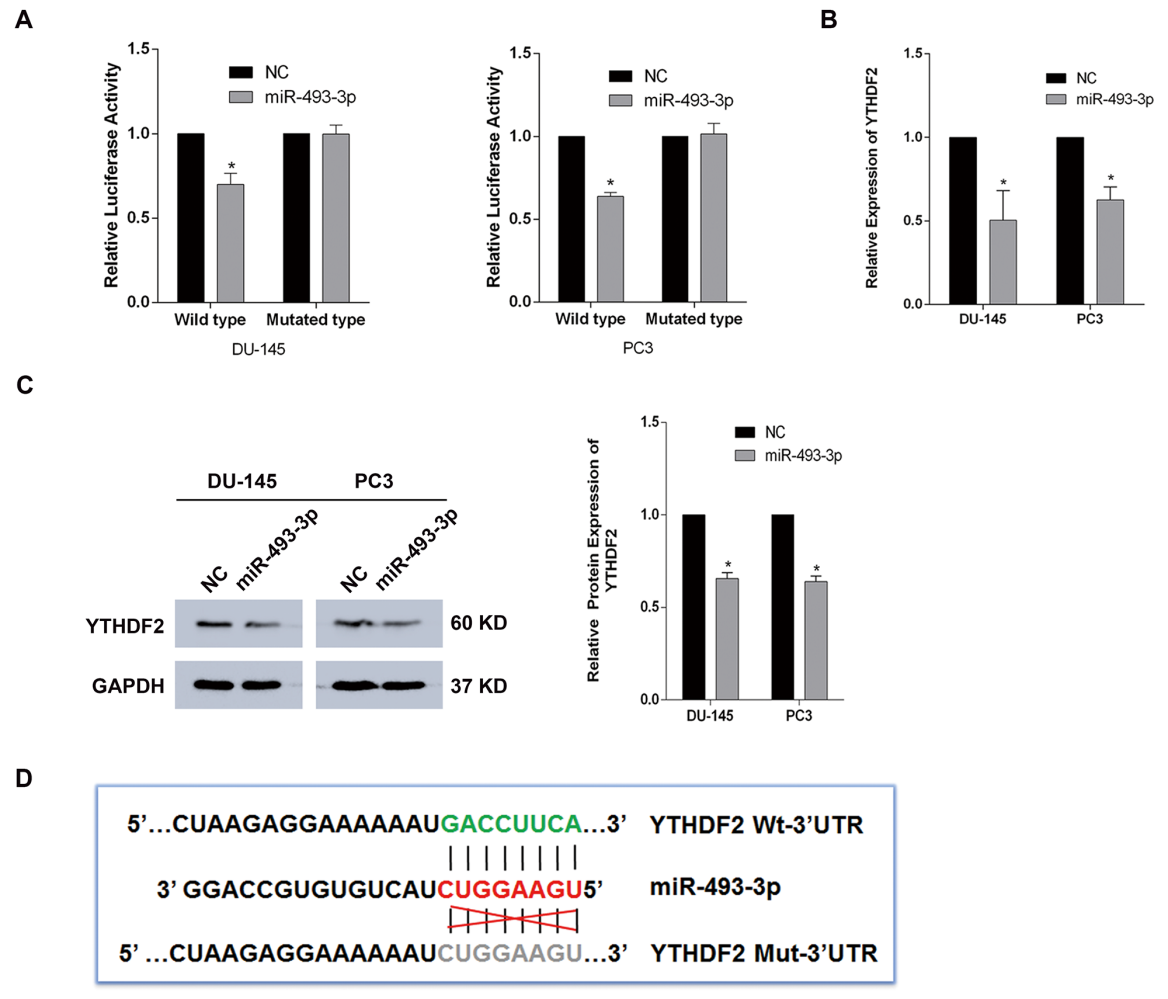
YTHDF2 is the direct target gene of miR-493-3p **(A)** Dual-luciferase reporter assay. miR-493-3p significantly reduced the luciferase activity of wild-type group but no significant reduction was observed in mutated-type group in both DU-145 and PC3 cell lines. **(B)** q-RT-PCR assay. The expression of YTHDF2 was significantly inhibited at mRNA level by overexpressed miR-493-3p. **(C)** Western blot assay. The expression of YTHDF2 was suppressed at protein level by miR-493-3p. And the band intensity was measured and the result was shown behind. **(D)** The schematic diagram showed the sequences of 3′-UTR of YTHDF2 (wild type and mutated type). Error bars represent the S.E. obtained from three independent experiments; ^*^; *P* < 0.05.

Based on several bioinformatics predictions (http://www.targetscan.org/, http://www.mirdb.org/) and the negative correlation between YTHDF2 and miR-493-3p in TMAs, we speculated that YTHDF2 may be the potential target of miR-493-3p. Dual-luciferase reporter assays were performed to determine whether miR-493-3p had a direct interaction with the 3′-UTR of YTHDF2. The results showed that, in both DU-145 and PC3 cell lines, the luciferase activity of wild-type group transfected with miR-493-3p compared with negative control was suppressed, whereas, no significant changes were observed in mutated-type group (Figure 3A). The q-RT-PCR and western blot assays were conducted to further determine alterations of the YTHDF2 at mRNA and protein levels. Consistently, overexpression of miR-493-3p downregulated the expression of YTHDF2 (Figure 3B and 3C). The sequences of the 3′-UTR of YTHDF2 (wild type and mutated type) were designed and presented as a schematic diagram (Figure 3D). To sum up, YTHDF2 is the direct target gene of miR-493-3p.

### Overexpression of miR-493-3p similarly elevates global mRNA m6A levels and inhibits cell proliferation and migration of PCa

**Figure 4:**
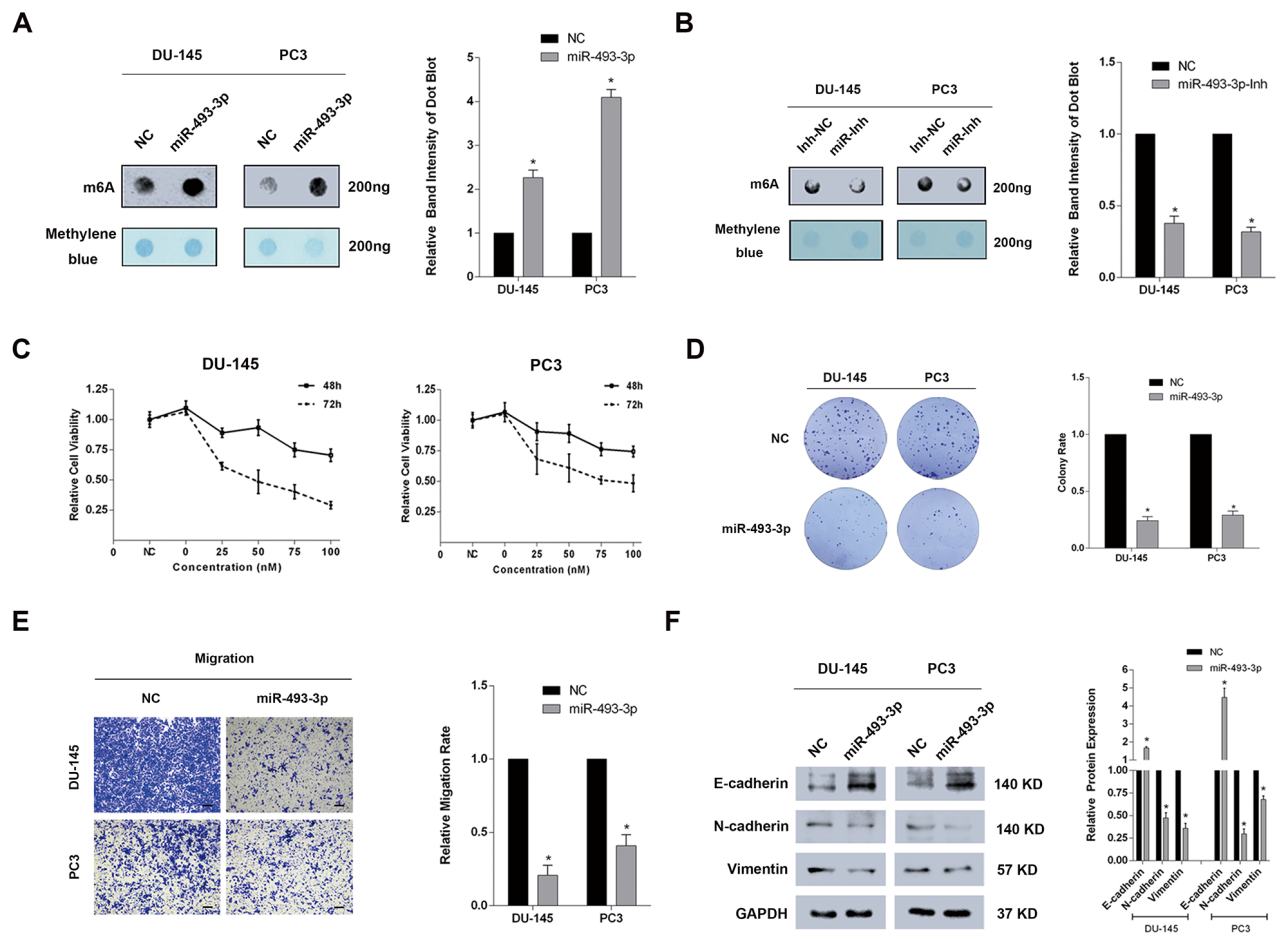
Forced expression of miR-493-3p significantly elevated the global m6A levels and inhibited cell proliferation and migration of PCa **(A)** m6A dot-blot assay. Forced expression of miR-493-3p significantly elevated m6A level in DU-145 and PC3 cell lines. And the band intensity was measured and the result was shown behind. **(B)** m6A dot-blot assay. Inhibition of miR-493-3p conversely reduced the m6A level in DU-145 and PC3 cell lines. And the band intensity was measured and the result was shown behind. **(C)** CCK-8 test. Overexpression of miR-493-3p significantly suppressed the proliferation at 48h and 72h in DU-145 and PC3 cell lines. **(D)** Colony formation assay. Overexpression of miR-493-3p significantly inhibited the colony formation ability in DU-145 and PC3 cell lines. And the migration rate was calculated and shown behind. **(E)** Trans-well assay. Overexpression of miR-493-3p inhibited the migration of DU-145 and PC3 cell lines. And the migration rate was calculated and shown behind. **(F)** Western blot assay. E-cadherin was upregulated, N-cadherin and Vimentin were downregulated in DU-145 and PC3 cell lines transfected with miR-493-3p mimics. And the band intensity of proteins was measured and the result was shown behind. Error bars represent the S.E. obtained from three independent experiments; ^*^; *P* < 0.05. Photographs of trans-well assay were obtained under 10x objective, and the scale bar = 100 μm.

Based on the above results that YTHDF2 was the target of miR-493-3p, m6A dot-blot was performed to investigate whether ectopic miR-493-3p could alter the global mRNA m6A levels. Interestingly, a significantly elevated m6A level was observed in both DU-145 and PC3 cell lines transfected with miR-493-3p mimics (Figure 4A). In contrast, inhibition of miR-493-3p reduced the global mRNA m6A levels (Figure 4B). To determine the function of miR-493-3p, a CCK-8 test was conducted and the results suggested the significant inhibition of proliferation at both 48h and 72h in two PCa cell lines (Figure 4C). A colony formation assay also indicated that overexpressed miR-493-3p mimics suppressed the colony formation rate in both cell lines. Furthermore, an obvious impairment of migration ability was detected in DU-145 and PC3 cell lines transfected with miR-493-3p (Figure 4E). Western blot assays revealed that miR-493-3p inhibited the migration by regulating EMT progression (Figure 4F). Collectively, forced expression of miR-493-3p also elevated the global mRNA m6A levels and inhibited cell proliferation and migration of PCa, which indicated the important role of miR-493-3p as a novel indirect-m6A regulator.

### Inhibition of miR-493-3p partially rescues si-YTHDF2-induced reduction of proliferation and migration in PCa

**Figure 5:**
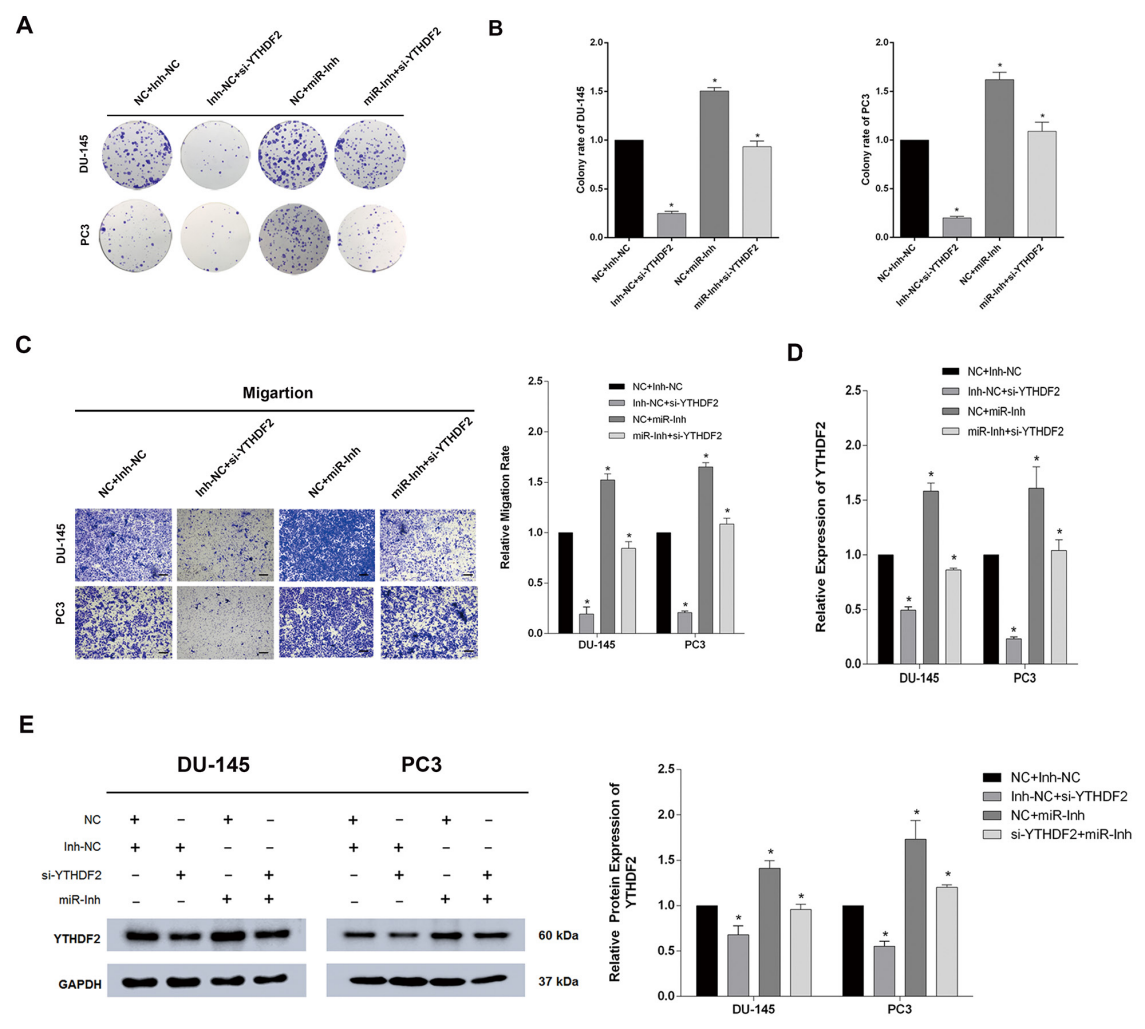
Inhibition of miR-493-3p partially abrogated the cell proliferation and migration suppressed by si-YTHDF2 **(A)** Colony formation assay. Inhibition of miR-493-3p reversed the colony formation ability repressed by si-YTHDF2. **(B)** The colony formation rate was calculated. **(C)** Trans-well assay. Inhibition of miR-493-3p reversed the migration repressed by si-YTHDF2. And the migration rate was calculated and shown behind. **(D)** and **(E)** q-RT-PCR and western blot. The expression of YTDHF2 at mRNA and protein levels were all detected a significant upregulation by inhibition of miR-493-3p. And the band intensity of proteins was measured and the result was shown behind. Error bars represent the S.E. obtained from three independent experiments; ^*^; *P* < 0.05. Photographs of trans-well assay were obtained under 10x objective, and the scale bar = 100 μm

To further confirm the direct interaction between YTHDF2 and miR-493-3p, a rescue experiment was applied to test whether inhibition of miR-493-3p could reverse the si-YTHDF2-induced phenotype. Interestingly, transfection of miR-493-3p-inhibitor (miR-Inh) significantly induced the colony formation, moreover, co-transfection of miR-493-3p-Inh and si-YTHDF2 partially abrogated si-YTHDF2-induced reduction of colony formation in DU-145 and PC3 cell lines (Figure 5A and 5B). Similarly, the migration ability investigated by trans-well assay, was also elevated after the inhibition of miR-493-3p, and a partial abrogation of si-YTHDF2-induced suppressed migration ability was also observed in DU-145 and PC3 cell lines (Figure 5C). The specific mRNA and protein levels of different transfected cells were all detected by q-RT-PCR and western blot assays, which revealed the a consistent change in level of YTHDF2 (Figure 5D and 5E). To conclude, rescue experiments confirmed the direct interaction between YTHDF2 and miR-493-3p, which were all involved in the m6A modification in PCa.

### Schematic diagram of YTHDF2 and miR-493-3p in modulating m6A modification of PCa

**Figure 6:**
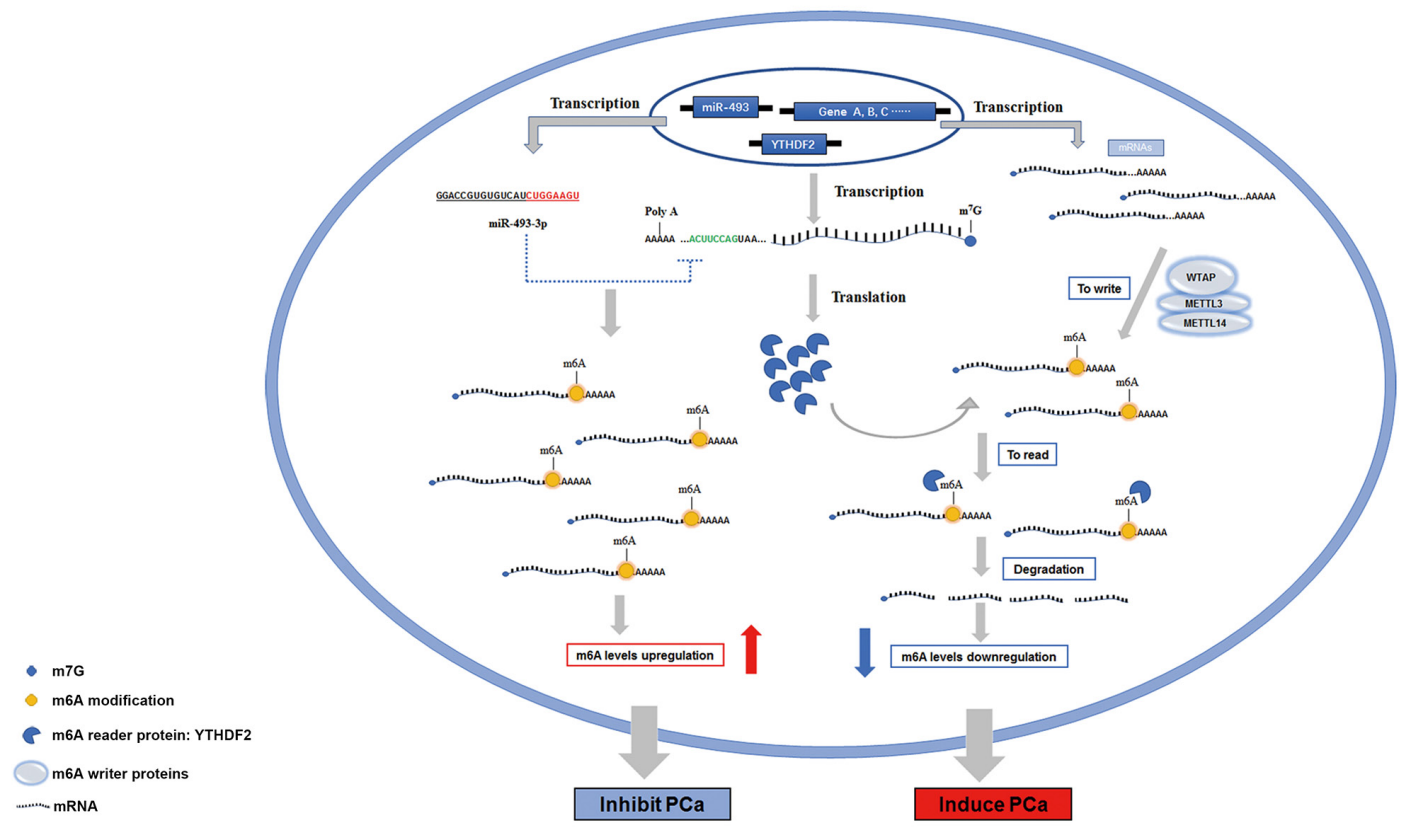
The schematic diagram of YTHDF2 and miR-493-3p in regulating the progression of PCa In PCa, total RNAs are transcribed and catalyzed by m6A writer (WTAP, METTL3 and METTL14) to produce the m6A modifications. YTHDF2, an m6A reader, recognizes and binds to the m6A modified sites to degrade the mRNAs, subsequently resulting in the reduction of the m6A levels, and consequently induced the progression of PCa. However, miR-493-3p transcribed from DLK1-DIO3 genomic region targets the 3’-UTR of YTHDF2 to suppress the translation of YTHDF2, thereby elevating the global RNAs m6A levels and inhibiting the progression of PCa.

Previously, the role of m6A modification in cancers were rarely studied, especially with regard to YTHDF2, its specific functions and mechanisms in PCa still remained elusive. Herein, we investigated the role of YTHDF2 and miR-449a in the regulation of PCa in m6A modification way. The specific mechanisms studied in this study are presented in a schematic diagram (Figure 6). All transcribed mRNAs were catalyzed by METTL3, METTL14 and WTAP complex at the N6-adenosine site. YTHDF2, as the m6A reader protein, degraded mRNAs and subsequently reduced the global mRNA m6A levels by binding to the m6A sites, which consequently induced the progression of PCa. However, overexpression of miR-493-3p suppressed the translation of YTHDF2 by targeting the 3’UTR, which inhibited the YTHDF2-induced m6A reduction and abrogated the progression of PCa at last. All these results indicated that m6A modification was significantly associated with the carcinogenesis of PCa. Furthermore, YTHDF2 and miR-493-3p acted as two crucial m6A regulators to be involved in the progression of PCa by indirectly regulating m6A levels.

## DISCUSSION

PCa is the most common cancer of men in developed countries, and holds the second highest mortality rate induced by cancer. However, the specific mechanisms of PCa are not fully elucidated [1]. m6A is a reversible post-transcriptional modification of RNA ‘written’ by a methyltransferase complex (METTL3, METTL14, WTAP and RBM15), ‘erased’ by demethylation complex (FTO and ALKBH5) and recognized and executed by various reader proteins (YTHDF1/2/3, YTHDC1/2, and HNRNPA2B1) in different ways [4-8]. So far, m6A modification has been reported to regulate mRNA splicing, export, and stability, and surprisingly, m6A modification has also been found to promote the translation of circular RNAs [12, 28-31]. Emerging evidence has indicated that the m6A modification is involved in tumor progressions. However, variable m6A levels occur in different cancers. The m6A modification in regulation of PCa is still poorly understood. In this study, we investigated how YTHDF2-mediated downregulation of m6A levels modulated PCa progression and how miR-493-3p serves as an indirect m6A regulator in above regulations.

YTHDF2, as a reader of m6A modification, belongs to a member of YTH domain family. YTHDF2 has been reported to trigger the degradation of targeted mRNAs and subsequently reduce the m6A level by recognizing and binding to m6A modified sites [21]. In addition, YTHDF2 can destabilize m6A modified RNAs through direct recruitment of the CCR4-NOT deadenylase complex [22]. Previous studies have revealed that YTHDF2 is involved in the development of AML [32]. Recently, YTHDF2 was found to regulate the m6A levels in HCC [23]. However, the function and mechanisms of m6A modification and YTHDF2 in PCa have not been elucidated so far. In this study, we detected that YTHDF2 was significantly upregulated in PCa cell lines and tissues compared with normal prostate epithelial cell line and adjacent normal tissues, which indicated the potential oncogene role of YTHDF2 in PCa. Further function studies confirmed that downregulation of YTHDF2 significantly inhibited the proliferation and migration of PCa cell lines. The m6A dot-blot assay was performed to detect the global mRNA m6A levels change in PCa cell lines treated with si-YTHDF2 or pYTHDF2. Interestingly, knock-down of YTHDF2 elevated the global mRNA m6A levels but overexpression of YTHDF2 reduced global RNA m6A level. Taken together, these data demonstrated that down regulation of YTHDF2 could inhibit the proliferation and migration by elevating global m6A levels.

miRNAs have been revealed to be involved in the development of various tumors by targeting the 3’-UTR of mRNAs. Similarly, several miRNAs have been identified and confirmed in PCa, including the onco-miRNAs (miR-21, miR-221, miR-222, miR-141, miR-375 and miR-18a etc.) and tumor suppressor miRNAs (miR-34a, miR-224, miR-452, miR-200b, miR-382, miR-372 etc.) [33]. However, the expression pattern and specific role of miR-493-3p in PCa still remains elusive. In this study, we found that miR-493-3p was significantly downregulated in PCa cell lines and tissues compared with the normal prostate epithelial cell line and adjacent normal tissues. Functional studies revealed that forced expression of miR-493-3p suppressed the cell proliferation and migration of PCa. Furthermore, statistical analysis showed a negative correlation between YTHDF2 and miR-493-3p, and dual-luciferase reporter assay confirmed YTHDF2 was a direct target of miR-493-3p. Interestingly, overexpression of miR-493-3p consistently elevated the global mRNA levels with knock-down of YTHDF2. A rescue experiment was conducted to further confirm the direct interaction between miR-493-3p and YTHDF2, and the results showed that inhibition of miR-493-3p could partially abrogate the suppression of proliferation and migration induced by si-YTHDF2. All above indicated that miR-493-3p was an indirect m6A regulator to inhibit the proliferation and migration by targeting YTHDF2, and both siYTHDF2 and miR-493-3p-increased methylated mRNAs may be also involved in the process of cell proliferation and migration in PCa.

In summary, we concluded the findings in this study as follows: (I) YTHDF2 is upregulated in both PCa cell lines and tissues compared with normal prostate epithelial cell line and adjacent normal tissues. (II) Knock-down of YTHDF2 significantly reduces the global mRNA levels and inhibited the proliferation and migration in PCa. (III) YTHDF2 is a direct target of miR-493-3p and negatively correlated with miR-493-3p. (IV) miR-493-3p is down regulated in PCa, and forced expression of miR-493-3p similarly elevated global mRNA m6A level and inhibited the proliferation and migration of PCa. (V) Inhibition of miR-493-3p partially abrogated the suppression of proliferation and migration induced by si-YTHDF2. (VI) Both YTHDF2 and miR-493-3p act as two crucial m6A regulators to indirectly regulate the progression of PCa in m6A way. Our study is the first to preliminarily evaluate the expression pattern, function and mechanisms of YTHDF2 in regulating PCa, and determine that miR-493-3p as the upstream factor of YTHDF2 is involved in the m6A modification and progression of PCa. We hope this study of YTHDF2 and miR-493-3p may provide new sights on the carcinogenesis and new potential therapeutic targets of PCa.

## MATERIALS AND METHODS

### Cell lines and cell culture

Human normal prostate epithelial cell line RWPE-1 and PCa cell lines DU-145 and PC3 were all obtained from the Cell Bank of Type Culture Collection of Chinese Academy of Sciences (Shanghai, China). All the cell lines were verified by short tandem repeat (STR) DNA profiling analysis. RWPE-1 cell line was cultured in K-SFM medium, and DU-145 and PC3 were cultured in MEM medium at 37°C under a humidified atmosphere of 5% CO_2_. Additional 10% heat-inactivated fetal bovine serum (FBS) was added to the above media.

### Chromogenic in situ hybridization (CISH)

Tissue microarrays (TMAs) were purchased from Xinchao Biotech, Shanghai, China, which contained 81 cases with paired tumor and non-tumor tissues. A 5′-DIG and 3′-DIG-labeled, locked nucleic acid-incorporated miRNA probe (miRCURY LNATM Detection probe, Exiqon, Woburn, MA, USA) was applied for the detection of miR-493-3p in PCa TMAs, The probe sequence of hsa-miR-493-3p was designed as follows: 5’-CCTGGCACACAGTAGACCTTCA -3’. The specific procedures were performed as previously described [34]. Both the intensity and proportion of positive cells were considered for the semi-quantification of the strength of positivity.

### Immuno-histochemical (IHC) staining

All paraffin tissue sections obtained from above TMAs were dewaxed and rehydrated. Antigen retrieval was performed by heating the slides in sodium citrate buffer (10 mM, pH 6.0). After blocking with bovine serum albumin (Sango Biotech, Shanghai, China), the slides were incubated with anti-YTHDF2 (Proteintech, Chicago, IL, USA) overnight at 4 °C. The slides were then incubated with a secondary antibody of goat antirabbit HRP conjugate (Cell Signaling Technology, Beverly, MA, USA) for 1 h at room temperature. A DAB solution was used for brown color development. HScores system was used to semi-quantify the strength of positivity which considered the intensity of the staining and the percentage of positive cells per the formula: HScores = 1 × (% light staining) + 2 × (% moderate staining) + 3 × (% strong staining), and HScores values range from 0 to 300.

### m6A dot-blot assay

To investigate the global mRNA levels, the m6A dot-blot assay was performed. Total RNAs were extracted from the transfected PCa cells with RNAiso plus (TaKaRa, Kusatsu, Japan), and the concentration of total RNAs were measured with Nano Drop, subsequently 1.8ug RNAs was isolated to prepare 50ng/ul (36ul) RNA solution. After the sufficient mix of 36ul RNA solution and 3 volume (108ul) RNA incubation buffer, 65°C incubation was performed to eliminate secondary structure. Cold isovolumetric 20× Saline-Sodium Citrate (SSC) Solution (Sigma-Aldrich, USA) was added to above treated RNA solution and maintained at 4°C. Washed with Milli-Q water and 10×SSC solution and dried, Amersham Hybond-N+ membrane (GE health, Chicago, USA) was stationed in bio-dot apparatus. After the wash (100ul Milli-Q water and 10×SSC solution) and water pump, 200ng (32ul) above pre-treated RNA was spotted to the membrane via bio-dot apparatus, followed by wash (100ul Milli-Q water and 10×SSC solution) and water pump again. After being dried, RNAs on the spotted membrane were UV cross-linked in a Ultraviolet Crosslinker. The cross-linked membrane was washed by Milli-Q water and incubated in 0.02% methylene blue (Sigma-Aldrich, USA) for 5 minutes. After incubation, several times of wash with Milli-Q water was conducted until the dyed background became diluted, then the photograph of dot methylene blue was obtained by scanning the membrane. The membrane was then washed three times with PBST for total 15 minutes, and blocked with 5 % non-fat milk for an hour. The fully blocked membrane was incubated with m6A antibody (Synaptic Systems, Goettingen, Germany) (1:2000) overnight. For the second day, after three times wash with PBST for total 15 minutes, the membrane was incubated with horseradish peroxidase (HRP)-conjugated anti-rabbit IgG secondary antibody. Another same wash was conducted and the membrane was visualized with Pierce™ ECL Western Blotting Substrate (Thermo, Massachusetts, USA).

### Dual-luciferase reporter assay

Oligonucleotide pairs possessing the potential desired miR-493-3p target region or mutant target region were all designed and purchased from Sangon, Shanghai, China. Double-stranded segments formed by the annealing step were inserted into pmirGLO Dual-Luciferase miRNA Target Expression Vector (Promega, Madison, WI, USA), between the SacI and SalI sites. Besides, the accuracy of the insertions were verified by sequencing. DU-145 and PC3 cell lines were seeded in 24-well plates and co-transfected with 50 nM miR-493-3p or NC and 100 ng reporter pmirGLO. The relative luciferase activity was measured by the Dual-Luciferase Reporter Assay System (Promega) 48 h after transfection.

### Transfection and reagents

All RNA duplexes were chemically synthesized by GenePharma Company (Shanghai, China). The corresponding sequences are listed as follows: NC (sense) 5’-ACTACTGAGTGACAGTAGA-3’, miR-493-3p (sense): 5’-UGAAGGUCUACUGUGUGCCAGG-3’, miR-493-3p-Inh (sense): 5’-CCUGGCACACAGUAGACCUUCA-3’, si-YTHDF2-1 (sense): 5’-GCCCAAUAAUGCAUAUACUTT-3’, si-YTHDF2-2 (sense): 5’-GCUCUGGAUAUAGUAGCAATT-3’, si-YTHDF2-3 (sense): 5’-GCGGGUCCAUUACUAGUAATT-3’. To achieve a better efficiency and avoid off-target effects, three si-RNAs were merged together to transfect PCa cell lines. All above RNA duplexes were transfected with Lipofectamine 2000 reagents (Invitrogen, Carlsbad, CA, USA) in accordance with the manufacturer’s protocol. And the specific transfection doses of miRNA, inhibitor, siRNA were maintained at 50nM concentration in all transfection experiments except the CCK-8 test. FuGENE HD Transfection Reagent (Promega, Madison, USA) was used to transfect the overexpressed plasmids according to the manufacturer’s protocol.

### Cell viability assay

When the seeded PCa cell lines grown to 30-50% of one well in 96-wells plates, the cells were treated with miR-493-3p mimics, miR-493-3p-Inh, si-YTHDF2-1/2/3 and pYTHDF2. Cell viability was examined by cell-counting-kit 8 (CCK-8) test, and the specific manipulations were conducted as before [34].

### Colony formation assay

DU-145 and PC3 cell lines transfected with miR-493-3p mimics or si-YTHDF2 for 48h were digested to seed in 6-well plates for 500 cells per well. After 2 weeks incubation at 37 under a humidified atmosphere of 5% CO_2_, the cells were fixed with methanol and stained with 0.3% crystal violet.

### Trans-well assay

ATrans-well assay was used to evaluate cell migration. Approximately 8×10^4^ DU-145 cells and PC3 cells (transfected with NC and mimics or siRNAs) suspended in 0.2 ml serum-free medium were placed onto the surface layer of the chamber. The entire chamber was placed into a 24-well plate, and 600 μL MEM medium supplemented with 10% FBS was added to the space between the chamber and the well. After incubation for 24 hours at 37 °C, we detected the migration rate using methanol and 0.3% crystal violet treatment. Photographs of trans-well assay were obtained under 10x objective, and scale bar = 100 μm.

### RNA isolation and qRT-PCR

RNA was extracted from PCa cell lines with RNAiso plus (TaKaRa, Kusatsu, Japan) and subsequently transcribed into cDNA with PrimeScript RT reagent Kit and a One Step PrimeScript miRNA cDNA Synthesis Kit. The expression levels of miRNA and mRNA were detected by qRT-PCR. SYBR Premix Ex Taq (TaKaRa, Kusatsu, Japan) was used to quantify the transcribed cDNA with the ABI 7500 fast real-time PCR System (Applied Biosystems, Carlsbad, CA, USA). Small nuclear RNA U6 and GAPDH mRNA were used as endogenous references to calculate the relative expression of associated genes with 2^-ΔΔCt^ (delta-delta-Ct algorithm) method. All primers used were listed in Supplementary Table 1.

### Western blot assay

Western blot analysis was performed as previously described [34]. The primary antibodies used in this study were listed as follows: anti-GAPDH, anti-YTHDF2, anti-Vimentin, anti-E-cadherin (Proteintech, Chicago, IL, USA), anti-N-cadherin (Cell Signaling Technology, Beverly, MA, USA).

### Statistical analysis

The data were expressed as the means ± S.D.. Differences between groups were estimated using the χ2-test or Student’s *t*-test. Pearson’s correlation coefficient was used to determine the correlation between the levels of miR-493-3p and YTHDF2 mRNA. All analyses were conducted using SPSS 16.0 software (IBM, Armonk, NY, USA), and significance was defined as a two-tailed value of p<0.05.

### Ethics approval and consent to participate

Approval to conduct this study was obtained from the Ethics Committees from the 1st Affiliated Hospital, College of Medicine, Zhejiang University, in accordance with the guidelines of the Helsinki Declaration of 1975, revised in 1983.

## SUPPLEMENTARY MATERIALS FIGURES AND TABLE


