# Supplementary material for: Downregulation of N6-methyladenosine binding YTHDF2 protein mediated by miR-493-3p suppresses prostate cancer by elevating N6-methyladenosine levels
Source: Oncotarget. 2017 Dec 18;9(3):3752–64. doi: 10.18632/oncotarget.23365 (PMC5790497; doi:10.18632/oncotarget.23365)
Supplement: Supplementary file 1 [file oncotarget-09-3752-s001.pdf]

Downregulation of N⁶-methyladenosine binding YTHDF2 protein mediated by miR-493-3p suppresses prostate cancer by elevating N⁶-methyladenosine levels

SUPPLEMENTARY MATERIALS

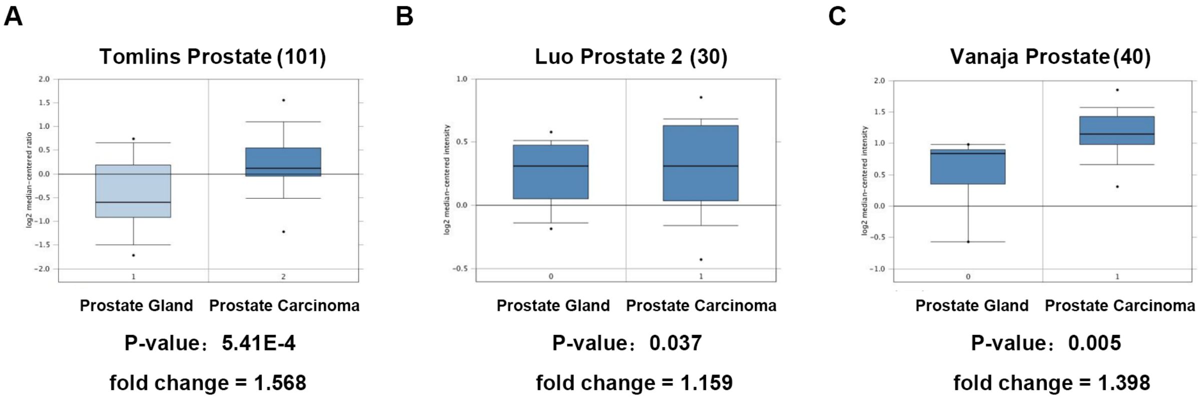

Supplementary Figure 1: The statistical analyses from three studies in Oncomine database: YTHDF2 was all upregulated in PCa. (A) Tomlins Prostate study, YTHDF2 is upregulated (FC = 1.568, P = 5.41E-4) in 101 PCa patients. **(B)** Luo Prostate 2 study, YTHDF2 is upregulated (FC = 1.159, P = 0.037) in 30 PCa patients. **(C)** Vanaja Prostate study, YTHDF2 is upregulated (FC = 1.398, P = 0.005) in 40 PCa patients.

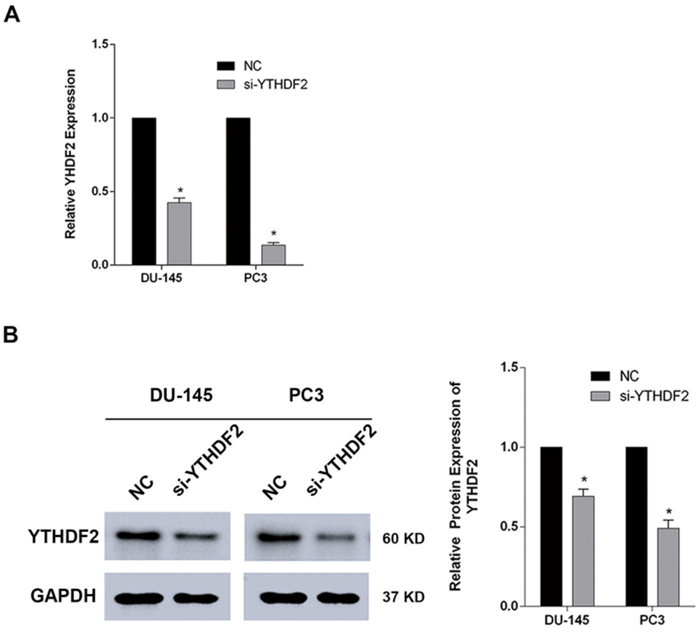

Supplementary Figure 2: The knock-down efficiency of si-YTHDF2-pool. (A) q-RT-PCR. Transfection with si-YTHDF2-pool significantly downregulate the expression of YTHDF2 at mRNA level. **(B)** Western blot. Transfection with si-YTHDF2-pool significantly downregulate the expression of YTHDF2 at protein level. And the band intensity of proteins was measured and the result was shown behind. Error bars represent the S.E. obtained from three independent experiments; $^{*}P < 0.05$.

Supplementary Table 1: The primers used in this study

Name[a]	Sequence(5′->3′)
YTHDF2-F	CCTTAGGTGGAGCCATGATTG
YTHDF2-R	TCTGTGCTACCCAACTTCAGT
GAPDH-F	AAGGTGAAGGTCGGAGTCA
GAPDH-R	GGAAGATGGTGATGGGATTT
miR-493-3p-F	TGAAGGTCTACTGTGTGCCAGG
U6-F	TGCGGGTGCTCGCTTCGGCAGC
YTHDF2-utr-wt-F	CATATCCTAAGAGGAAAAAATGACCTTCAAGAGAATTAGGACTTTTTTCG
YTHDF2-utr-wt-R	TCGACGAAAAAAGTCCTAATTCTCTTGAAGGTCATTTTTTCCTCTTAGGATATGAGCT
YTHDF2-utr -mut-F	CATATCCTAAGAGGAAAAAATCTGGAAGTAGAGAATTAGGACTTTTTTCG
YTHDF2-utr-mut-R	TCGACGAAAAAAGTCCTAATTCTCTACTTCCAGATTTTTTCCTCTTAGGATATGAGCT

a: F, forward primer; R, reverse primer.

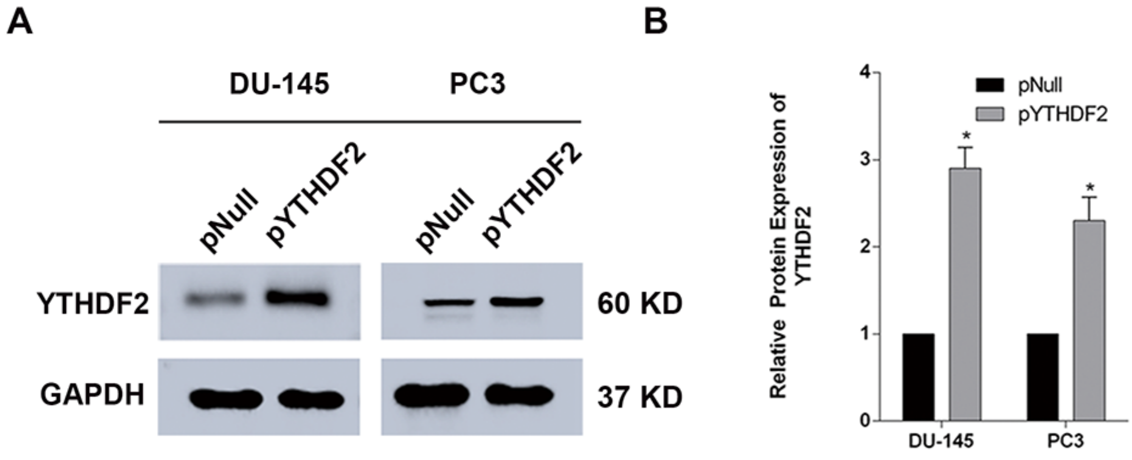

Supplementary Figure 3: The overexpression efficiency of pYTHDF2. **(A)** Western blot showed a significant upregulation of YTHDF2 at protein level by transfecting with pYTHDF2. **(B)** The band intensity of proteins was measured. Error bars represent the S.E. obtained from three independent experiments; $^{*}P < 0.05$.
